# Transcriptome analysis of the production enhancement mechanism of antimicrobial lipopeptides of *Streptomyces bikiniensis* HD-087 by co-culture with *Magnaporthe oryzae* Guy11

**DOI:** 10.1186/s12934-022-01913-2

**Published:** 2022-09-10

**Authors:** Wei Liu, Jiawen Wang, Huaqian Zhang, Xiaohua Qi, Chunmei Du

**Affiliations:** 1 Engineering Research Center of Agricultural Microbiology Technology, Ministry of Education, Heilongjiang University, Harbin, 150500 Heilongjiang China; 2 Key Laboratory of Microbiology, College of Heilongjiang Province, School of Life Sciences, Heilongjiang University, Harbin, 150080 Heilongjiang China

**Keywords:** Co-culture, Lipopeptide, Transcriptome, *Streptomyces bikiniensis*, *Magnaporthe oryzae*

## Abstract

The lipopeptides produced by *Streptomyces bikiniensis* have a significant inhibitory effect on *Magnaporthe oryzae*, but the low yield limits its application. In this study, the anti-*M. oryzae* activity of the broth of *S. bikiniensis* HD-087 co-cultured with *M. oryzae* Guy11 mycelium has risen by 41.22% compared with pure culture, and under induction conditions of adding Guy11-inducer (cell-free supernatant of *M. oryzae* Guy11), the activity of strain HD-087 improved 61.76%. The result proved that the enhancement effect of Guy11 on the antimicrobial activity of HD-087 was mainly related to metabolites but mycelium cells. Under optimum induction conditions, *NRPS* gene expression levels of HD-087 were significantly increased by induction with Guy11-inducer, the biomass of HD-087 had no significant change, but crude extract of lipopeptide (CEL) production was 107.4% higher than pure culture, and TLC result under acid hydrolysis showed that the induced culture has one component more than pure culture. To clarify the regulation mechanism of improving lipopeptide production of HD-087 with Guy11-inducer, transcriptomic analysis was performed using RNAseq to compare the induced culture and pure culture. In the induced culture, 943 genes were up-regulated, while 590 genes were down-regulated in DEGs (differentially expressed genes). KEGG results showed that the expression of genes related to amino acid synthesis, fatty acid metabolism, TCA cycle and pyruvate metabolism pathway were significantly increased. The increased expression of genes related to these metabolic pathways provided sufficient precursors for lipopeptide synthesis. Accordingly, key enzyme genes responsible for the synthesis of lipopeptides *Srf* and *NRPS* was significantly increased. Quorum sensing related genes *OppA* and *MppA* were significantly up-regulated, and then *ComP* was activated and promoted lipopeptide synthesis. These results provided a scientific basis for using *M. oryzae* to induce the increase of the production of *Streptomyces* lipopeptides, and also laid a foundation for further exploring the co-culture mechanisms among different genera.

**Supplementary Information:**

The online version contains supplementary material available at 10.1186/s12934-022-01913-2.

## Introduction

Rice (*Oryza sativa* L.) is among the most important food crops in the world, and blast disease caused by *Magnaporthe oryzae* is one of the most devastating diseases [1]. Currently, rice blast control strategies rely on the application of chemical fungicides, the use of resistant varieties, and crop rotation [2]. However, with the increase in the number of years of chemical fungicide application, the continuous increase in pathogen resistance to fungicides and the accumulation of chemical residues have evolved into problems that can not be ignored, in addition to the increasing cost of using chemical fungicides [3]. Biological control of plant diseases is known to be more cost-effective, safe and environmentally friendly than fungicides [4]. *Streptomyces* and their metabolites have long been an important resource for the biological control of plant diseases, and their beneficial ecological effects are related to their ability to produce a wide range of antimicrobial compounds, including non-peptide compounds such as polyketides, amino glycans, phospholipids, and peptide compounds such as cyclic lipopeptides (CLPs). CLPs are cyclic peptides with amino acid residues and fatty acid tails of varying lengths, which are synthesized through nonribosomal peptide synthetase [5]. This large antimicrobial group is composed of three main families: fengycins, iturins and surfactins [6]. This amphiphilic structure modifies surface tension of cell membranes allowing pore formation, ultimately, triggering cell apoptosis [7]. However, the high production cost and low yield of lipopeptides have limited their large-scale industrial production and application. So, improving the yield of lipopeptides has been the center of attention. To this day, lipopeptide production has been successfully increased by promoter engineering [8], rewiring regulatory networks [9], heterologous expression [10] or co-culture [11].

It should be noted that co-culture strategies have proven simple and efficient without the need for complex genetic level operations, expensive reagents and have been widely applied in the field of discovering novel and bioactive microbe-derived natural products [12]. Co-culture mimicking the natural environment through mixed fermentation of different microorganisms (also called co-cultivation) may lead to an enhancement in the production of compounds [13] and even trigger the expression of silent biosynthetic pathways [14], leading to the accumulation of new natural products [15]. Posada Uribe indicated that a change in available space, nutrients, pH, light, and/or oxygen occurs with the presence of a nearby competitor (such as a plant pathogen), which introduces significant environmental stress on the CLP-producing microorganism [16]. Defilippi et al. [3] also analyzed the CLP yield changes during the co-culture of *Bacillus subtilis* B9-5 with *Fusarium sambucinum*, *Verticillium dahliae* and *Rhizopus stolonifer*. It was reported that co-culture with the moderately sensitive fungus *F. sambucinum* and tolerant fungus *R. stolonifer* produced a large number of CLP during the interactions [3]. Therefore, it is feasible to use plant pathogenic fungi to induce the increase of lipopeptide production and develop new lipopeptide by induced co-culture modelling.

Our previous studies have shown that *S. bikiniensis* HD-087 has antioxidant activity against cucumber *Fusarium wilt* and *F. oxysporum* [17], and its metabolites contain lipopeptide, which significantly inhibit the growth of *M. oryzae* mycelium and reduce the disease index of *M. *oryzae ([18, 19]. To improve the production of lipopeptides in HD-087, in this study, the co-culture of *M. oryzae* Guy11 and *S. bikiniensis* HD-087 enhanced the production of lipopeptides and produced a new peptide compared to pure culture. Transcriptomics was used to reveal further possible regulatory mechanisms to increase the production of lipopeptides. Therefore, it provides a new idea to produce lipopeptide by co-culture fermentation.

## Materials and methods

### Test strains and culture medium

*Streptomyces bikiniensis* HD-087 was isolated, screened, identified and preserved by the microbiology laboratory of Heilongjiang University. *Magnaporthe oryzae* Guy11 was donated by Dr. Chong Zhang of Shenyang Agricultural University. Gauze’s synthetic broth medium No. 1 [20] was used for strain HD-087 activation, DBY medium [19] was used for HD-087 fermentation for lipopeptide production, and PDB medium [21] was used for Guy11 culture.

### Preparation of lipopeptide crude extract

*S. bikiniensis* HD-087 was inoculated in Gauze’s synthetic broth medium No. 1 and cultured in a shaking incubator at 180 r·min^−1^ and 28 °C for 3 d. Then, 2 mL of culture solution was inoculated into a 250 mL flask containing 50 mL of DBY medium and incubated on a shaker at 180 r·min^−1^ for 4 d at 28 °C for containing the fermentation broth. Crude extract of lipopeptide (CEL) was obtained from the fermentation broth by acid precipitation and alcohol extraction according to the method of Gong [22].

### Co-culture conditions of *S. bikiniensis *HD-087 and *M. oryzae* Guy11

Inoculation strategies of the co-culture group were carried out as follows: firstly, 2 mL of HD-087 seed liquid was inoculated into a 250 mL flask containing 60 mL of DBY medium, then different quantity (0.1 g, 0.2 g and 0.3 g separately) of Guy11 mycelium were inoculated into DBY medium at different time including simultaneous inoculation (without delay) and sequential inoculation (inoculated with a delay of 12 h, 24 h with respect to the inoculation of HD-087), separately (The treatment serial number was recorded with inoculation time and inoculation quantity, for example, 0–0.1 g means the treatment was inoculated 0.1 g M*. oryzae* mycelium without delay, and so on). All inoculation treatments were incubated on a shaker at 180 r·min^−1^ for 4 d at 28 °C, and fermentation broth was collected. The anti-*M. oryzae* activity of different fermentation broths were determined by measuring the dry weight of *M. oryzae* mycelium [23] and the cup and saucer method [24] at 200 μL, and equivalent sterile water was used as a control.

### The preparation of Guy11 cell-free supernatant and the induction conditions

*M. oryzae* Guy11 was cultured in PDB medium as specified above conditions, then the broth was centrifuged at 9,500 r·min^−1^ and filtrated with bacterial filter to obtain the culture supernatant as the Guy11-inducer. And then, inoculation strategies of the induced culture group were carried out as follows: first, 2 mL of HD-087 seed liquid was inoculated into a 250 mL flask containing 60 mL of DBY medium, then different volumes (1 mL, 2 mL and 3 mL separately) of Guy11-inducer were inoculated into DBY medium at a different time (inoculation time; culture conditions and anti-*M.oryzae* activity methods shown in 2.3), equivalent PDB medium was used as a control (The treatment serial number was recorded as 2.3, 0–1 mL means inoculating 1 mL Guy11-inducer without delay, and so on).

### Real-time fluorescence quantitative PCR

Total RNA was extracted from three treatments using Trizol (Beyotime Biotechnology Co., LTD., Shanghai, China), namely, pure culture, the 24–0.1 g which had the best effect against *M. oryzae* in co-culture group and the 24–1 mL which was selected for the same reasons from induced culture). The cDNA template was synthesized by reverse transcription using a kit (Vazyme Biotechnology Co., LTD., Nanjing, China). The qPCR was performed using SYBR green on a 7500 Fast Real-time PCR System (Applied Biosystems, Foster, CA, USA). The primers were designed according to the gene sequences from *S. bikiniensis* HD-087 complete genome, with 16S rRNA as reference gene and synthesized by Sangon Biotechnology Co. LTD. (Shanghai, China). Real-time fluorescence quantitative PCR was performed with the following protocol: Pre-denaturation at 95.0 °C for 30 s; 39 cycles of denaturation at 95.0 °C for 30 s, annealing at 57.0 °C for 20 s and extension at 72 °C for 15 s (the primers were shown in Additional file 1: Table S1).

### CLE yield and biomass assay in optimum induction conditions

In optimum induction conditions of Guy11-inducer on HD-087, the CEL dry weight was measured and calculated after extraction by acid precipitation and alcohol, with pure culture as control. Biomass of *S. bikiniensis* HD-087 mycelium was determined by the method of Liu et al., and pure culture as control [25].

### Component analysis by thin layer chromatography (TLC)

Two activated silica gel GF_254_ thin layers were taken and labeled as plate A and plate B. Diluted CELs of the best co-culture and pure culture with methanol to the concentration of 10 mg/mL, then took 10 μL by capillary tube for sampling. After spreading with chloroform/methanol/water (65:25:4) and drying, plate A was directly sprayed with 0.5% ninhydrin reagent for color development. Plate B was placed in an airtight container in which a small beaker containing 2 mL hydrochloric acid was put in advance and fumigated at 110 °C for 3 h. After cooling and blowing away hydrochloric acid, 0.5% ninhydrin solution was sprayed to develop color.

### Transcriptome analysis

After 72 h of culture, HD-087 hyphae ball was sampled separately from the best co-culture and pure culture broth at the same growth period, then sent to Sangon Biotechnology Co. LTD. (Shanghai, China) for transcriptome sequencing (using Illumina sequencing system). The gene expression of exons per thousand bases per million pieces mapping (FPKM) value was calculated by using the DESeq software according to the following conditions-statistical significant differences in the detection of gene expression: | log_2_ (fold) |> 1.0 and p-values < 0.05. Differentially expressed genes (DEGs) were functionally classified by gene ontology (GO) enrichment analysis and Kyoto Encyclopedia of Genes and Genomes (KEGG) pathway analysis.

### Data analysis

All the above experiments were repeated three times with consistent data. Data from representative samples were analyzed by one-way analysis of variance (ANOVA) followed by means separation using the least significance difference (LSD) test at a significance of P < 0.05.

## Results

### Comparative analysis of anti-*M. oryzae* activity between pure culture and co-culture

**Fig. 1:**
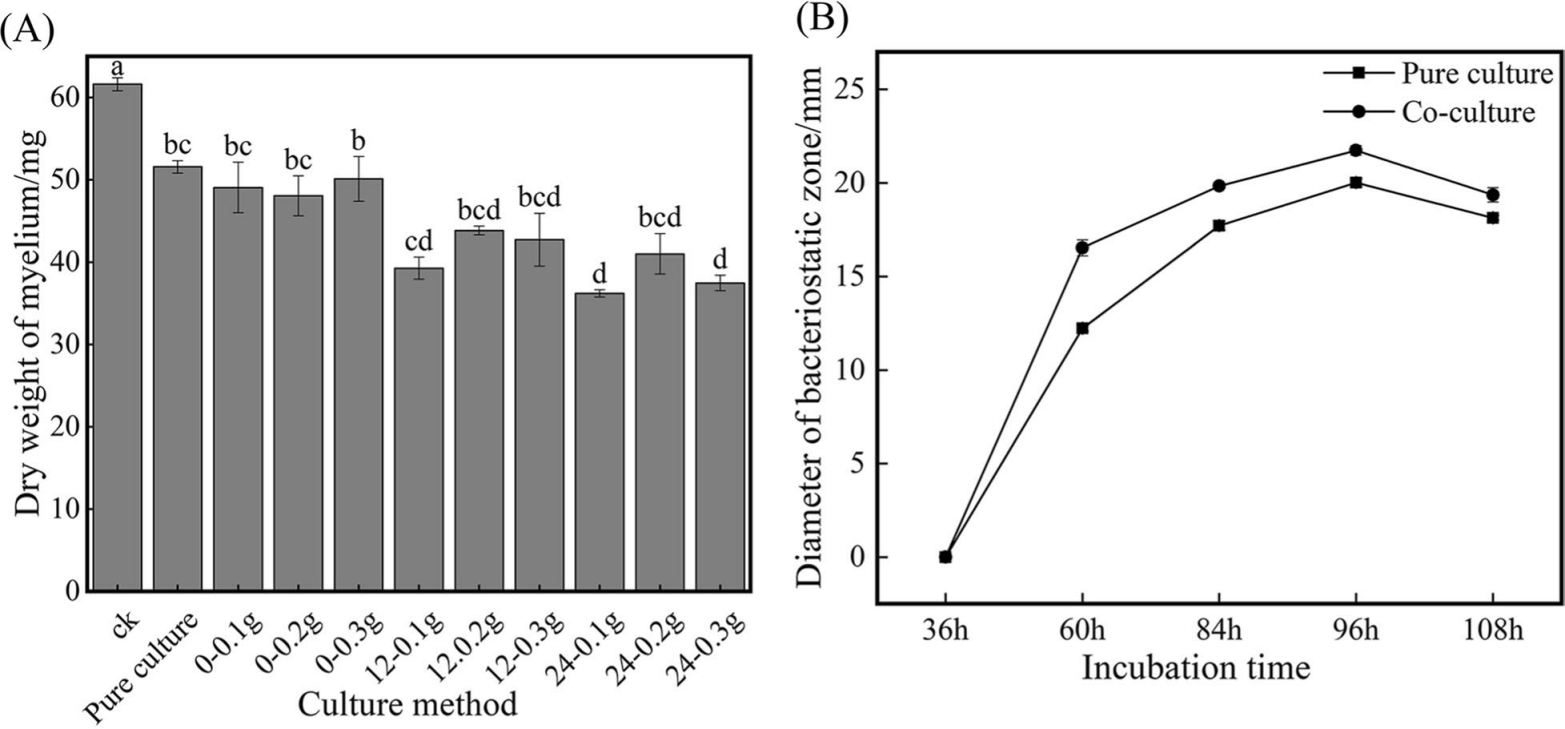
The inhibition effect of different broths obtained by co-culture and pure culture on the growth of *M. oryzae* **A** Effects on the dry weight of *M. oryzae* mycelia; **B** Effects on the diameter of the inhibition zone against *M. oryzae*. Different small letters indicated significant different among samples (P < 0.05)

To investigate the effect of *M. oryzae* Guy11 on the production of anti-*M. oryzae* substance of *S. bikiniensis* HD-087, the co-culture of Guy11 and HD-087 with different inoculations was carried out. According to Fig. 1A, when Guy11 and HD-087 seed liquids were simultaneously inoculated, the anti-*M. oryzae* activity of the co-culture broth was the minimum, but with the delay of inoculating Guy11, the antifungal effect of the fermentation liquid showed an increasing trend. When 0.1 g Guy11 mycelium was added 24 h later (24–0.1 g), the inhibition effect of co-culture broth on *M. oryzae* growth was the strongest, and the inhibition rate of the dry weight of *M. oryzae* mycelia was 41.22%. Under this inoculation condition, the anti-*M. oryzae* activity of co-culture at different times compared to pure culture as shown in Fig. 1B, the inhibition effect of co-culture supernatant was up to the highest at 96 h, the diameter of inhibition zone was 21.74 ± 0.26 mm, which was 8.6% higher than that of pure culture. The results showed that *M. oryzae* Guy11 could induce *S. bikiniensis* HD-087 to produce more antimicrobial metabolites.

### Comparative analysis of anti-*M. oryzae* activity between pure culture and add the inducer

**Fig. 2:**
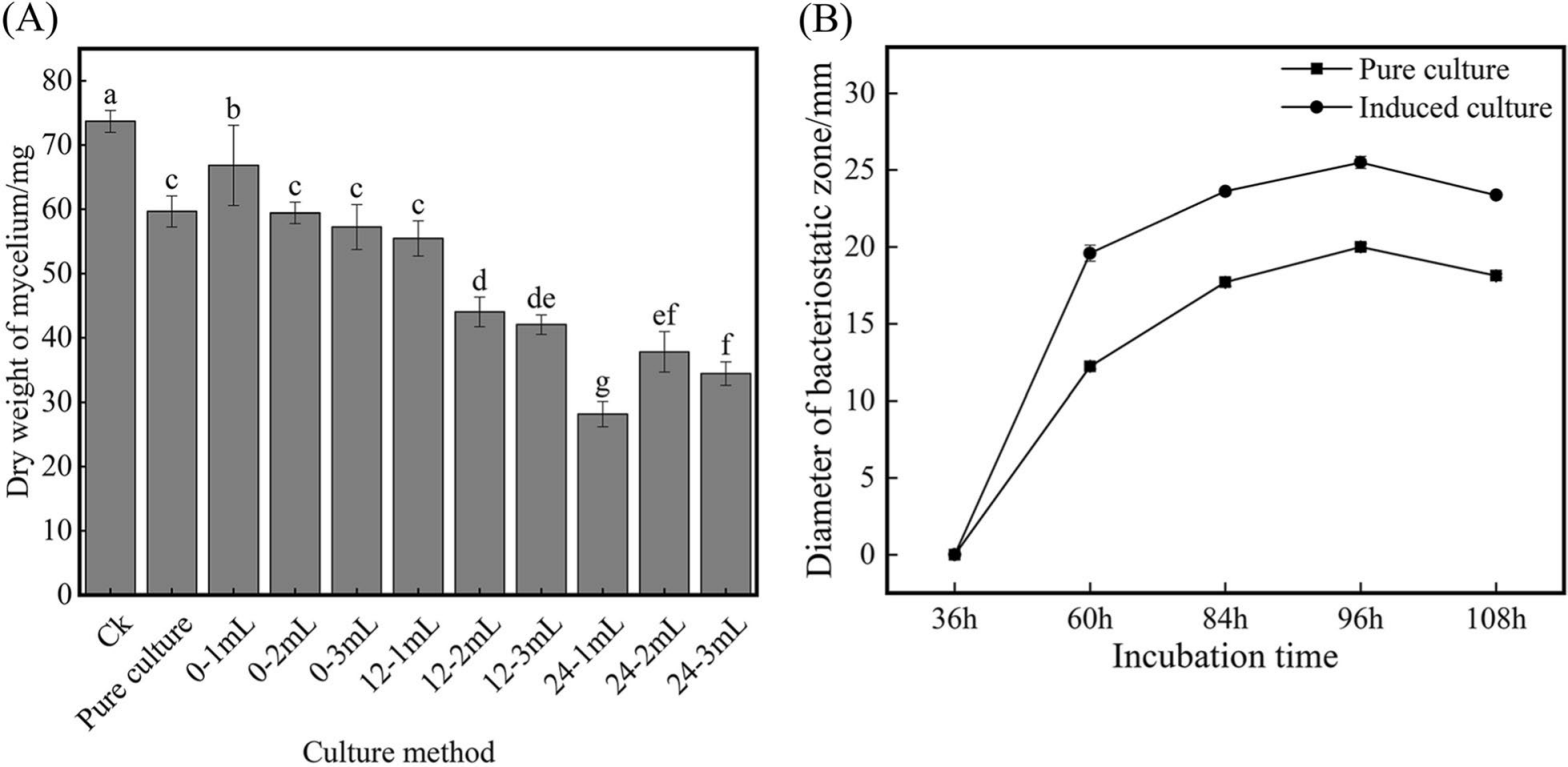
The inhibition effect of different broths obtained by induced culture and pure culture on growth of *M. oryzae.* **A** Effects on the dry weight of *M. oryzae* mycelia; **B** Effects on the diameter of the inhibition zone against *M. oryzae*. Different small letters indicated significant difference among samples (P < 0.05)

To investigate whether the enhancement effect of *M. oryzae* on *S. bikiniensis* HD-087 is cell-dependent, the Guy11-inducer (cell-free) was used to stimulate HD-087. Results as shown in Fig. 2A. When 1 mL inducer was added 24 h later (24–1 mL), the stimulation effect was the strongest, and the fermentation broth of HD-087 showed the best inhibition effect on the mycelium growth of *M. oryzae*. The dry weight of mycelium of *M. oryzae* is significantly lower than those of CK (water), pure culture and the other inducer groups, and the inhibition rate was up by 61.76% over pure culture. As can be seen from Fig. 2B, the anti-*M.oryzae* effects of the 24–1 mL induced group always significantly superior than that of pure culture at all test times for the bigger bacteriostatic zone. Especially, when the total culture time was 96 h (at this time HD-087 had been induced by Guy11*-*inducer for 72 h), the bacteriostatic zone diameter was the biggest (25.5 ± 0.40 mm) and 22.0% higher than that of the pure culture. Obviously, it can be inferred that *S. bikiniensis* HD-087 in induction culture should be induced to produce more anti-*M. oryzae* substances by the inducer.

### NRPS gene expression by RT-PCR

**Fig. 3:**
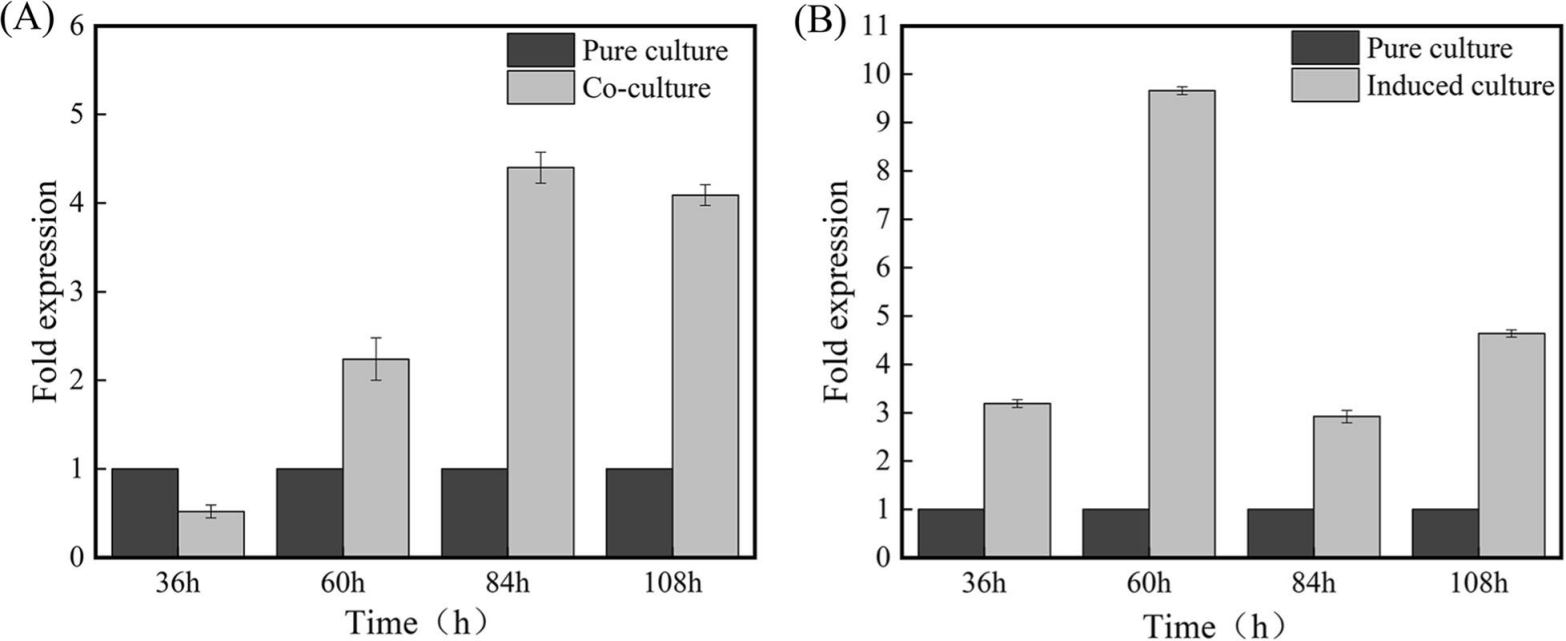
*NRPS* gene expression in different culture groups **A** Compared co-culture with pure culture; **B** Compared induced culture with pure culture

As shown in Fig. 3A, the *NRPS* gene in co-culture group (24–0.1 g) decreased at 36 h compared with pure culture group, which may be due to the interaction between *M. oryzae* Guy 11 and *S. bikiniensis* HD-087 in early stage, and reduced the biological activity of HD-087. After that, HD-087 produced a stress response, and the *NRPS* gene significantly increased until 108 h and the highest expression level was recorded at 84 h, which was 4.66 times higher than pure culture group. As shown in Fig. 3B, *NRPS* gene in the induced group was significantly increased at each time due to the existence of Guy11-inducer (24-1 mL), and the expression level of the *NRPS* gene was highest at 60 h, which appeared 12 h earlier than co-culture and was 9.66 times than pure culture group.

### Biomass analysis and yield estimation

**Fig. 4:**
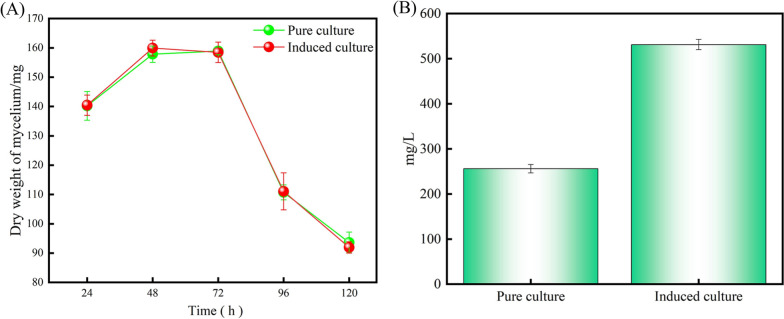
Comparative analysis of biomass and yield of CEL obtained by induced culture and pure culture **A** Lipopeptide production; **B** Lipopeptide yield

The biomass results showed that there was no remarkable change in the biomass under optimum induction conditions (Fig. 4A). Then we measured the change in lipopeptide production, the results were shown in Fig. 4B. The lipopeptide production of the induced group was 531.3 ± 9.3 mg/L, and 107.4% higher than pure culture.

### TLC analysis of CEL

**Fig. 5:**
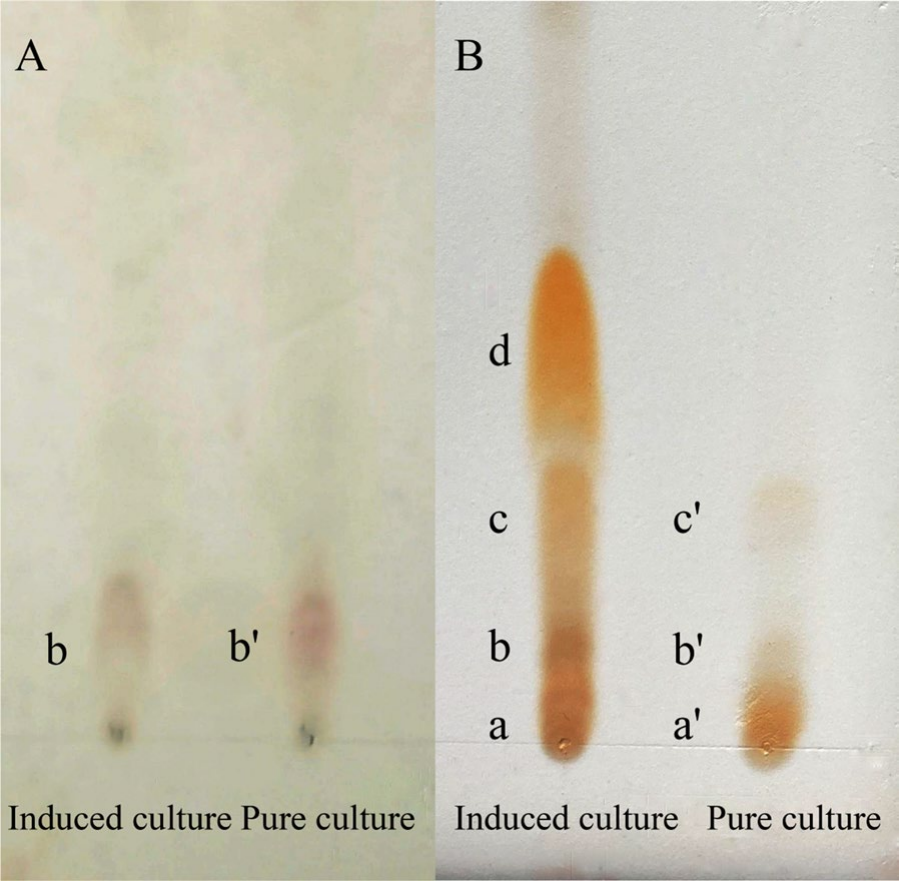
TLC result of lipopeptide extract. Note: plate A was directly sprayed with ninhydrin for color rendering; plate B was sprayed with ninhydrin after it was acid hydrolyzed with ninhydrin in situ

TLC results were shown in Fig. 5, both induced culture and pure culture only appeared with one purplish-red spot in plate A, which indicated that non-closed linear peptide or free amino acid were exists in both groups. The color spots of plate B increased and appeared orange, indicating the presence of closed cyclic peptide that developed color after high-temperature acidolysis, and in the location of induced culture, there was one more orange spot than in pure culture. The result illustrated that HD-087 produce a new component of peptide under the conditions of stimulating with *M. oryzae* Guy11 inducer compared with pure culture. At the same time, the color of spots b and c of induced culture was darker, indicating that CLP content at the two spots was higher than that of pure culture.

### Transcriptome analysis

#### Illumina sequencing assembly data quality analysis

Six cDNA libraries from induced culture and pure culture were sequenced to study the transcriptomes of *S. bikiniensis* HD-087 induced by *M. oryzae* Guy11-inducer. Adaptors and low-quality sequences were screened from the original data, and the average was cleaned for reading. After mapping to the reference genome (Table 1), all Q_20_ and Q_30_ values of the read sequences in the samples exceeded 98% and 93%, respectively.

**Table 1:** Summary of transcriptome sequencing data of *S.bikiniensis* HD-087

Sample	Raw data(bp)	Clean reads(bp)	Q20%	Q30%
Pure culture	4,912,665,600	31,697,352	98.96%	95.68%
Induced culture	7,314,554,700	46,688,486	98.57%	93.65%

#### Identification of DEG

**Fig. 6:**
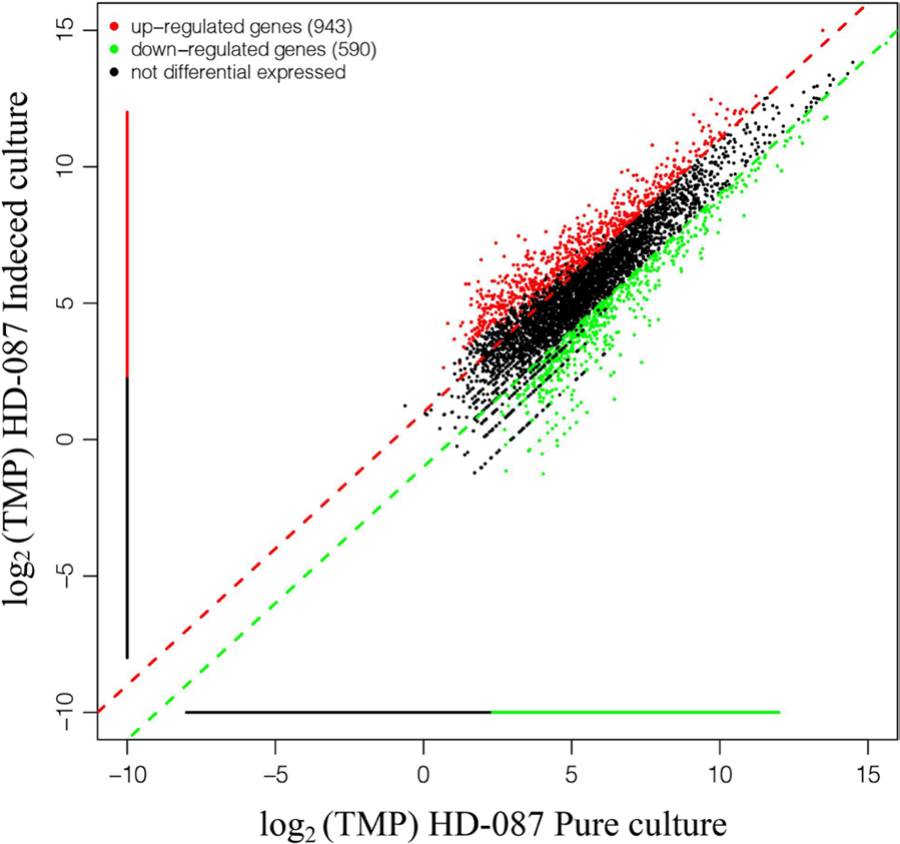
Volcano plot of DEGs between induced culture and pure culture

1533 differentially expressed genes (DEG) were detected between induced culture and pure culture, located in | log2 (fold) | > 1.0 and p-values < 0.05. Compared with pure culture, 943 genes were significantly up-regulated and 590 genes were significantly down-regulated in induced culture (Fig. 6).

#### DEG was functionally classified by GO and KEGG pathway analysis

**Fig. 7:**
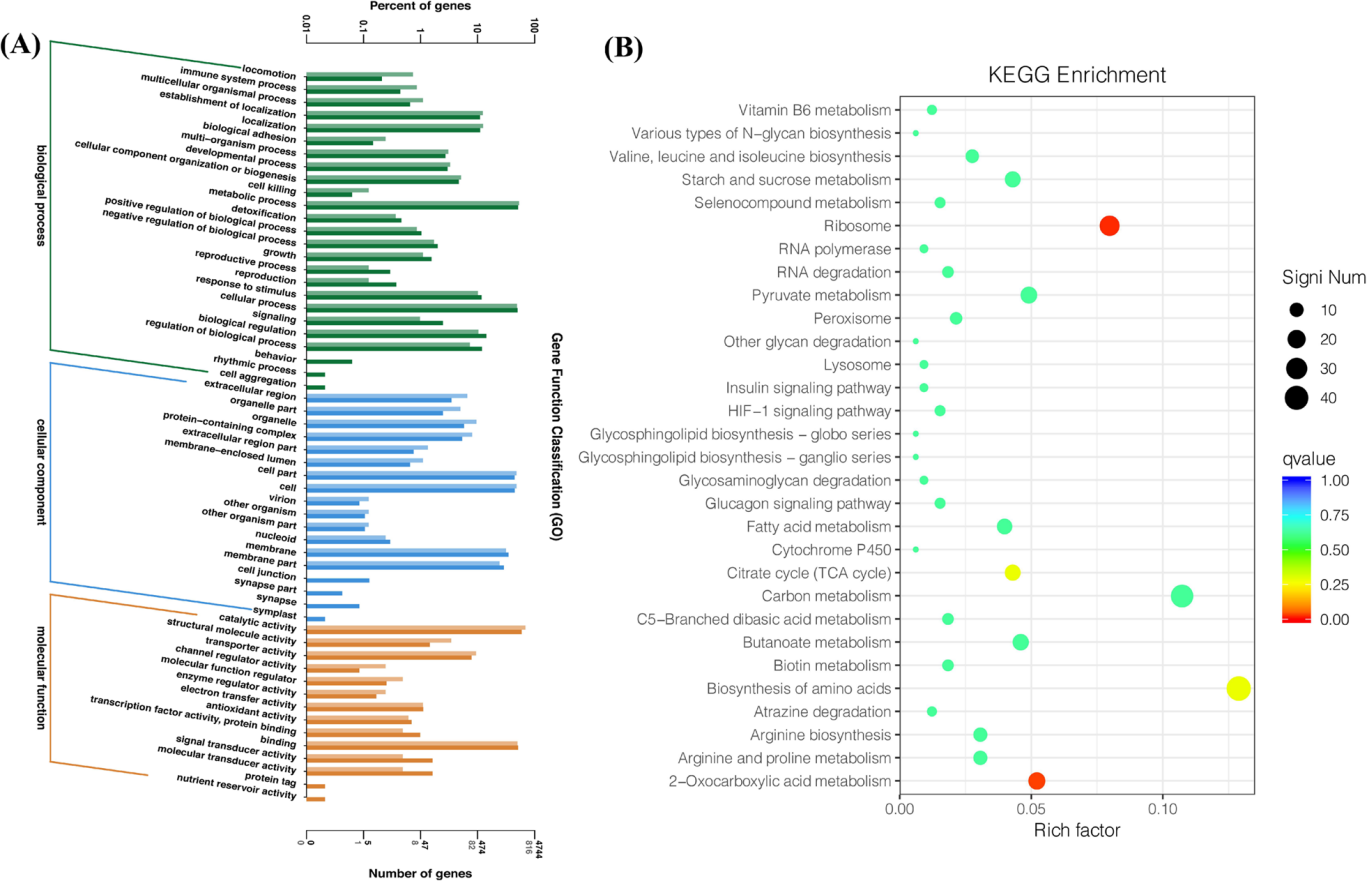
GO enrichment and DEGs KEGG enrichment analysis.** A** Gene ontology (GO) enrichment analysis of the differently expressed genes at induced culture compared with pure culture;** B** KEGG analysis of up-regulated genes in *S. bikiniensis* HD-087 transcriptome in induced culture and pure culture

To investigate the molecular changes of *S.bikiniensis* HD-087 under the influence of *M. oryzae* Guy11-inducer, GO and KEGG pathways were used to analyze DEGs and their main biological functions were determined. The enrichment genes in the three GO categories were summarized in Fig. 7A. In the cellular component category, the GO terms are mainly enriched in the cell; cell part; membrane and membrane part. Similarly, in the molecular function category, binding; catalytic activity and transporter activity were significantly enriched. Further, in the biological process cellular process, metabolic process; biological regulation and regulation of biological process were significantly enriched.

The biological functions associated with DEGs were further analyzed using the KEGG database. It was found that mainly DEGs occurred in many metabolisms. Such as arginine and proline metabolism, arginine biosynthesis, atrazine degradation, amino acid biosynthesis and metabolism of biotin, butyric acid metabolism, C5 branched diacid metabolism, carbon metabolism, TCA cycle, cytochrome P450, fatty acid metabolism, glucagon signaling pathways, sugar glycosaminoglycans degradation, sheath sugar lipid biosynthesis, the festival series, sheath sugar, lipid biosynthesis-ball series, HIF-1 signaling pathways, insulin signaling pathway, lysosome, other chitosan degradation, peroxidase, pyruvate metabolism, RNA degradation, RNA polymerase, ribosomes, selenium metabolism, starch and sucrose metabolism, valine, leucine and isoleucine biosynthesis, various types of N-chitosan creatures synthesis, vitamin B6 metabolism and other pathways (Fig. 7B).

#### Analysis of genes and pathways related to lipopeptide synthesis

**Fig. 8:**
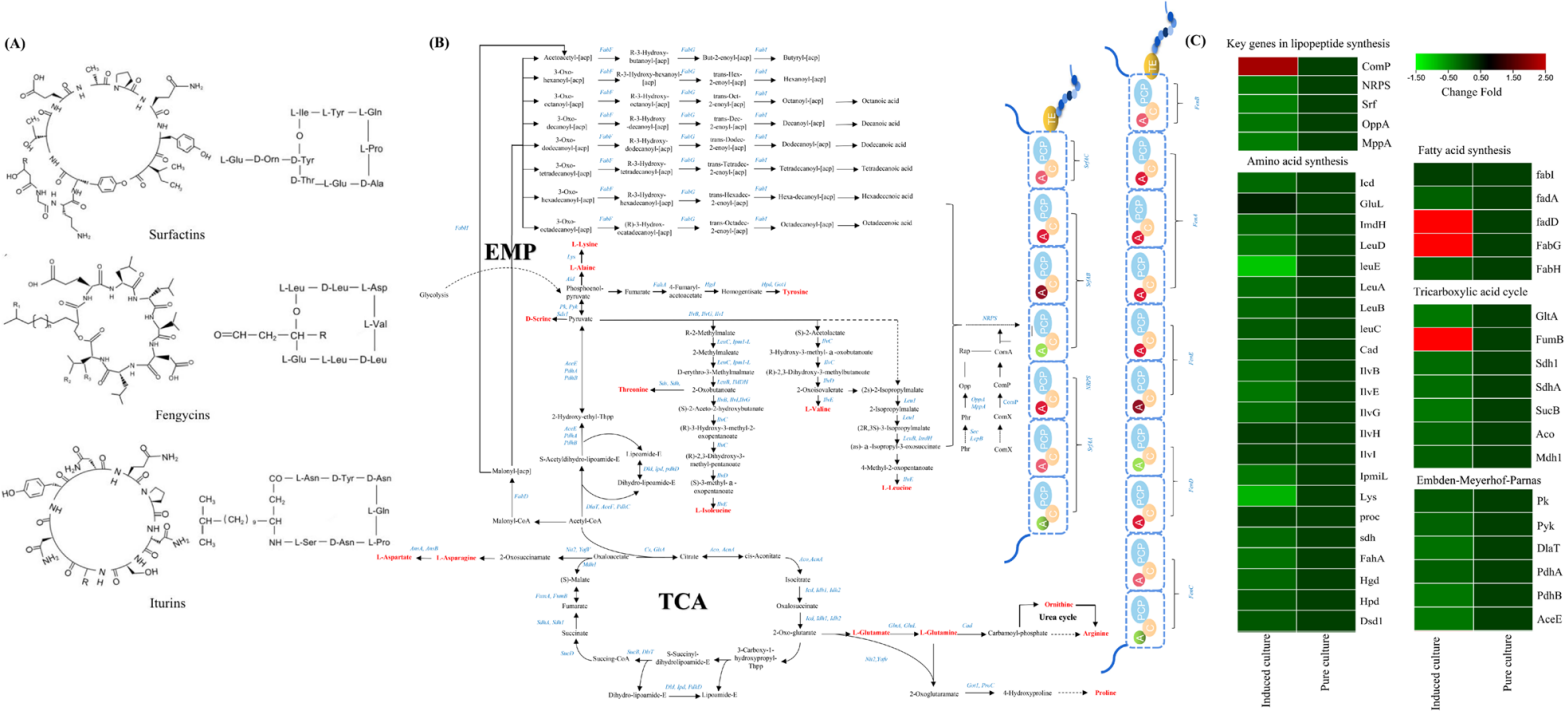
Effect of induced culture on expressions of genes involved in lipopeptide synthesis. **A** Chemical structure and amino acid composition of lipopeptide; **B** Genes related to the response to *M. oryzae* inducer treatment(lipopeptide related amino acids are shown in red font); **C** Genes heatmap with significantly up-regulated and down-regulated expression is compared with the pure culture

Further analysis was carried out according to amino acid structure, lipopeptide composition (Fig. 8A) and lipopeptide synthesis pathway. The expression of genes related to amino acid biosynthesis, fatty acid metabolism, TCA cycle and pyruvate metabolism pathway changed under the effect of *M. oryzae* Guy11-inducer (Fig. 8B). There were twenty genes significantly up-regulated in amino acid synthesis pathway and five genes significantly up-regulated in fatty acid synthesis pathway. Six genes were significantly up-regulated in the pyruvate pathway. Seven genes were significantly up-regulated in the TCA cycle. Under induced conditions (Guy11-inducer existing), Quorum sensing related genes *OppA* (peptide ABC transporter substrate-binding protein), *MppA* (ABC transporter permease) were significantly up-regulated, and then *ComP* was activated, leading to up-regulation of lipopeptide synthesis genes ( *NRPS and Srf),* and jointly promote the enhancement of lipopeptide synthesis (Fig. 8C and Additional file 2: Table S2). Additionally, we also found that many genes were up-regulated but did not reach | log2 (fold) |> 1.0, including *Fen*, *Cs*, *LepB* and other important genes related to lipopeptide synthesis. Some key genes were not found to be silenced or down-regulated in all down-regulated genes. The down-regulated genes are mainly in the pentose phosphate pathway, biosynthesis of pentose and glucuronate interconversion and non-lipopeptide amino acids.

## Discussion

At present, co-culture fermentation has been applied in many metabolite production processes. For example, the co-culture fermentation of *Streptomyces rochei* MA37 and *Pseudomonas* sp. (without direct contact, but allowing substance and signal exchange) led to an up-regulation of the production of several metabolites. In addition, the expression of the cryptic indole alkaloid BGC in MA37 was induced [26]. *S. rochei* MB037 and *Rhinocladiella similis* 35 co-cultures not only produced two new fatty acids borrelidin J and K with a rare nitrile group, but also the production of 7-methoxy-2, 3-dimethylchromone-4-one was significantly increased [27]. The marine fungus *Cosmospora* sp. and *M. oryzae* co-culture induce five soudanones A, E, D and two new derivatives, soudanones H and I [15]. However, no report has been found on the use of *M. oryzae* to stimulate or induce *Streptomyces* for increasing CLP production by co-culture or induced culture.

In this study, *M. oryzae* Guy11 mycelium can stimulate *S. bikiniensis* HD-087 to improve the antifungal activity when both were co-cultured, and the antifungal activity of HD-087 was set off more remarkable by Guy11-inducer (cell-free supernatant of *M. oryzae* Guy11) than co-culture. It was found that the enhanced effect of HD-087 on resistance to *M. oryzae* was mainly related to the metabolites in Guy-inducer but mycelium cells. Under induced conditions, the mycelium biomass of HD-087 did not changed, but the lipopeptide yield significantly increased. TLC analysis showed that the induced group produced a new peptide component compared to pure culture. But the result didn't means a new compound which has not yet been discovered. Of course, there is such a possibility that the compound is unique, further NMR or LC–MS analysis is necessary to confirm it. Increasing lipopeptide yield and producing new components mainly depended on some simulation factors in Guy11-inducer. Transcriptomes in pure culture and induced culture were analyzed to elucidate the mechanism of Guy11-inducer stimulating lipopeptide production. The results showed that the TCA cycle, EMP pathway, amino acid metabolism and fatty acid synthesis pathways of key upstream processes of lipopeptide synthesis were significantly changed in induced culture.

Among them, The enhancement of TCA cycle could produce more NADH and FADH2 (transferred to ATP by oxidative phosphorylation), intermediates for other cellular physiological processes such as carbon fixation, nitrogen assimilation, and fatty acid biosynthesis [28] and fatty acid synthesis is a key step for lipopeptide synthesis. The TCA cycle produce more ATP and pyruvate, where pyruvate could be converted into acetyl-CoA to participate the TCA cycle, amino acids synthesis, and lipid metabolism [29, 30]. *Fum B*, *Mdh1*, *Aco*, *Suc* and other genes of TCA ring were significantly up-regulated, providing material and energy basis for the synthesis of lipopeptides. Correspondingly, gene expressions of leucine, valine, glutamate and proline biosynthesis in amino acid metabolic pathways in induced culture were significantly increased and they are important precursors of lipopeptide synthesis. These constituent amino acids are assembled through the NRPS multi-enzyme complex, comprising adenylation, condensation, and thiolation domains responsible for the activation of amino acids and peptide chain elongation [31]. Therefore, the up-regulated expression of *NRPS* genes in induced culture is the key factor to improve lipopeptides production. The enhancement of *Srf* expression shifted the metabolic flow at acetyl CoA node from cell growth to surfactin biosynthesis, and the production of surfactin was further improved. Surfactin has been mainly reported for its resistance to bacteria and can significantly enhance other antifungal effects. However, in recent years, some reports have shown that surfactins also have antifungal activity against *Fusarium oxysporum*, *F. moniliforme*, *F. solani* [32], *Candida albicans* [33] and so on. The expression of *Fen* gene was also increased in induced culture, which was 1.76 times higher than pure culture. Fengycin has significant antifungal activity against other pathogens [34]. Surfactin and fengycin B could exhibit a synergistic inhibitory effect on *Phytophthora infestan* [35]. Consequently, the up-regulated expression of surfactin and fengycin genes were presumed to be an important factor for the improvement of anti-*M. oryzae* activity of induced culture/co-culture broth. In other words, the increase of lipopeptide yield by induced culture/co-culture should attribute to the activation of lipopeptide biosynthetase gene by *M. oryzae* inducer.

The expression of *FabD*, *FabH*, *FabG* and *FabI* genes related to fatty acid biosynthesis were all up-regulated in induced culture by induction of *M. oryzae* inducer. The FabD (acyl carrier protein) S-malonyl transferase is an enzyme containing malonyl-CoA and acyl carrier protein substrates, whereas they are CoA and malonyl-acyl-carrier-protein. The transfer of malonate to acyl-carrier-protein (ACP) converts the acyl groups into thioester forms which are characteristic of acyl intermediates in fatty acid synthesis and strictly required for the condensation reactions catalyzed by β-ketoacyl-ACP synthetase [36]. The FabH was considered to catalyze the first elongation reaction (Claisen condensation) of type II fatty acid synthesis, resulting in the production of short-chain fatty acid primers [37]. The action of β-ketoacyl-acyl carrier protein (ACP) synthase III (FabG) condenses these branched acyl-CoAs with malonyl-ACP. At the same time, malonyl-acyl carrier protein (ACP) is catalyzed to condense with acetyl -CoA to form β -ketobutyyl-ACP, which is the precursor of straight-chain fatty acids [38]. Therefore, the up-regulated expression of fatty acid synthesis genes indicates that *S. bikiniensis* HD-087 can be induced to produce a large number of lipopeptides under the stimulation from *M. oryzae* Guy11*-*inducer.

In the quorum sensing pathway, *OppA and MppA* genes were up-regulated significantly, and the OPP protein increased, meanwhile, *Sec* and *LepB* genes were up-regulated in some degree, helps to transport pheromone Phr and positively regulated OPP. The OPP negatively regulates Rap protein, and then Rap negatively regulate ComA, and thus ComA enhances lipopeptide synthesis. Furthermore, Guy11-inducer significantly activated the expression of *ComP,* then ComP interact with ComA and improved lipopeptide synthesis. This result revealed that the quorum-sensing related genes were closely related to lipopeptide synthesis. Bendori also reported that the oligopeptide signaling molecule ComX and pheromone Phr jointly regulate the expression of lipopeptide synthesis gene and Phr peptide inhibits the activity of co-transcribed Rap protein [39]. Thus the presence of the inducer stimulated some quorum-sensing gene expression, and then promoted the gene expression of lipopeptide biosynthetic enzymes, and ultimately improved the lipopeptides production of *S. bikiniensis* HD-087.

Although the construction of artificial co-culture system is relatively simple but metabolic regulation is relatively complex [40]. Therefore, a detailed analysis of the related pathways of lipopeptides is very important to better understand the increase production mechanisms of lipopeptides. These results explained the molecular mechanism of lipopeptide yield enhancement of *S. bikiniensis* HD-087 by stimulating with inducer (the culture supernatant of *M. oryzae* Guy11), and illustrated the relationship of the up-regulating genes involved in lipopeptide synthesis. Moreover, to some extent, using a microbial metabolite to induce lipopeptide production enhancement is easier to control in the fermentation process and downstream post-treatment process. This study provides a scientific basis for improving the yield of lipopeptide by inducing culture.

## Conclusion

In this study, we determined the two culture methods (co-culture and induced culture) can promote/enhance the production of antimicrobial lipopeptides of *S. bikiniensis* HD-087. It was found that the anti-*M. oryzae* activity of induction culture was enhanced more significantly and a new component of peptide observed compared to pure culture in lipopeptide extract of *S. bikiniensis* HD-087 in TLC. The biomass of HD-087 mycelium between pure culture and induced culture had no remarkable difference, the enhancement of lipopeptide production was caused by some metabolite (maybe some signal molecule) in Guy11-inducer. Transcriptome analysis showed Guy11-inducer promoted high-efficiency expression of the TCA cycle, EMP pathway, amino acid synthesis pathway, fatty acid synthesis pathway-related genes and key enzyme genes *NRPS*, *Srf* and quorum sensing related gene *comP* of *S. bikiniensis* HD-087. This provides a scientific basis for improving the yield of lipopeptide by co-culture.

## Supplementary Information

**Additional file 1: Table S1.** Primers needed for fluorescence quantification PCR.

**Additional file 2: Table S2.** Effect of induced culture on expressions of genes involved in lipopeptide synthesis with the log_2_FC values of genes (FC = fold change)

## Data Availability

All datasets contained in this study are listed in the manuscript.
