# Supplementary material for: Transcriptome analysis of the production enhancement mechanism of antimicrobial lipopeptides of Streptomyces bikiniensis HD-087 by co-culture with Magnaporthe oryzae Guy11
Source: Microb Cell Fact. 2022 Sep 10;21:187. doi: 10.1186/s12934-022-01913-2 (PMC9464393; doi:10.1186/s12934-022-01913-2)
Supplement: Supplementary file 1 — Additional file 1: Table S1. Primers needed for fluorescence quantification PCR. [file 12934_2022_1913_MOESM1_ESM.docx]

Table S.1 Primers needed for fluorescence quantification PCR

| Gene name | Primer sequence |
| --- | --- |
| *16sRNA* | 5^，^-TAACCCAACATCTCACGACAC-3^，^ |
|  | 5^，^-CGCAAGGCTAAAACTCAAAG-3^，^ |
| *nrps* | 5^，^-CCGCAGGTCTCCTTCAACTA-3^，^ |
|  | 5^，^-TCGGAGTAGAACCAGGTGAAG-3^，^ |
