# Supplementary material for: Transcriptome analysis of the production enhancement mechanism of antimicrobial lipopeptides of Streptomyces bikiniensis HD-087 by co-culture with Magnaporthe oryzae Guy11
Source: Microb Cell Fact. 2022 Sep 10;21:187. doi: 10.1186/s12934-022-01913-2 (PMC9464393; doi:10.1186/s12934-022-01913-2)
Supplement: Supplementary file 2 — Additional file 2: Table S2. Effect of induced culture on expressions of genes involved in lipopeptide synthesis with the log2FC values of genes (FC = fold change) [file 12934_2022_1913_MOESM2_ESM.docx]

Table S2. Effect of induced culture on expressions of genes involved in lipopeptide synthesis with the log_2_FC values of genes (FC = fold change).

| Gene ID | Symbol | log_2_FC | p-Value | Annotation |
| --- | --- | --- | --- | --- |
| amino acid biosynthes | | | | |
| 5650 | Icd | 1.53 | 4.36×10^-96^ | isocitrate dehydrogenase, NADP-dependent |
| 5584 | GluL | 3.95 | 3.09×10^-15^ | Glutamine synthetase |
| 4734 | ImdH | 1.61 | 6.97×10^-26^ | 3-isopropylmalate dehydrogenase |
| 1520 | LeuD | 1.05 | 1.02×10^-10^ | 3-isopropylmalate dehydratase small subunit |
| 4270 | leuE | -1.79 | 7.34×10^-13^ | leucine efflux protein LeuE |
| 1567 | LeuA | 1.43 | 2.87×10^-6^ | (R)-citramalate synthase |
| 1573 | LeuB | 1.58 | 7.11×10^-31^ | 3-isopropylmalate dehydrogenase |
| 1521 | leuC | 2.06 | 8.25×10^-106^ | 3-isopropylmalate dehydratase large subunit |
| 5763 | Cad | 1.66 | 3.26×10^-6^ | carbamoyl-phosphate synthase small subunit |
| 1590 | IlvB | 1.39 | 1.00×10^-12^ | acetolactate synthase large subunit |
| 1572 | IlvE | 1.07 | 3.4×10^-10^ | branched chain amino acid aminotransferase |
| 5703 | IlvG | 1.41 | 1.10×10^-6^ | acetolactate synthase large subunit |
| 1589 | IlvH | 3.01 | 2.77×10^-28^ | acetolactate synthase small subunit |
| 0535 | IlvI | 2.86 | 2.52×10^-8^ | acetolactate synthase small subunit |
| 5145 | IpmiL | 1.07 | 5.33×10^-6^ | isoleucine--tRNA ligase |
| 1775 | Lys | -1.07 | 4.67×10^-10^ | diaminopimelate decarboxylase |
| 3804 | proc | 2.79 | 1.27×10^-5^ | pyrroline-5-carboxylate reductase |
| 2993 | sdh | 1.57 | 8.56×10^-2^ | succinate dehydrogenase |
| 3950 | FahA | 1.20 | 6.70×10^-3^ | fumarylacetoacetase |
| 5475 | Hgd | 1.60 | 6.59×10^-5^ | homogentisate 1,2-dioxygenase |
| 5619 | Hpd | 2.20 | 6.92×10^-17^ | 4-hydroxyphenylpyruvate dioxygenase |
| 4384 | Dsd1 | 2.11 | 9.31×10^-5^ | amino acid deaminase |
| fatty acid metabolism | | | | |
| 5363 | fabI | 2.95 | 1.58×10^-11^ | Enoyl-[acyl-carrier-protein] reductase |
| 0931 | fadA | 1.82 | 5.08×10^-12^ | acetyl-CoA acetyltransferase |
| 5492 | fadD | 16.59 | 3.71×10^-3^ | acyl-CoA synthetase |
| 3887 | FabG | 16.77 | 9.55×10^-3^ | beta-oxoacyl-ACP reductase |
| 0201 | FabH | 2.08 | 6.57×10^-3^ | 3-oxoacyl-ACP synthase |
| TCA cycle | | | | |
| 2605 | GltA | 1.01 | 1.23×10^-3^ | citrate synthase/methylcitrate synthase |
| 4435 | FumB | 17.61 | 2.34×10^-6^ | hypothetical protein |
| 4257 | Sdh1 | 1.43 | 2.38×10^-7^ | succinate dehydrogenase |
| 4255 | SdhA | 1.38 | 5.93×10^-37^ | succinate dehydrogenase flavoprotein subunit |
| 5036 | SucB | 1.08 | 2.92×10^-29^ | 2-oxoglutarate dehydrogenase, E2 component, dihydrolipoamide succinyltransferase |
| 0946 | Aco | 1.672 | 2.02×10^-112^ | aconitate hydratase |
| 4204 | Mdh1 | 1.86 | 3.40×10^-179^ | malate dehydrogenase |
| pyruvate metabolism pathway | | | | |
| 5212 | Pk | 2.16 | 7.13×10^-65^ | pyruvate kinase |
| 5213 | Pyk | 1.72 | 5.14×10^-4^ | hypothetical protein |
| 0627 | DlaT | 1.22 | 8.60×10^-2^ | peptide ABC transporter substrate-binding protein |
| 3212 | PdhA | 1.87 | 3.59×10^-9^ | pyruvate dehydrogenase (acetyl-transferring) E1 component subunit alpha |
| 3226 | PdhB | 1.04 | 5.21×10^-4^ | pyruvate dehydrogenase (acetyl-transferring) E1 component subunit alpha |
| 3225 | AceE | 1.02 | 1.20×10^-2^ | 2-oxoisovalerate dehydrogenase |
| lipopeptide synthesis | | | | |
| 0498 | ComP | 14.86 | 9.15×10^-3^ | Quorum-Sensing |
| 3737 | NRPS | 1.55 | 3.12×10^-5^ | non-ribosomal peptide synthetase |
| 3735 | Srf | 1.12 | 4.41×10^-5^ | non-ribosomal peptide synthetase |
| 2010 | OppA | 1.63 | 6.05×10^-9^ | peptide ABC transporter substrate-binding protein |
| 2009 | MppA | 1.20 | 3.36×10^-54^ | ABC transporter permease |
